# Expression Profile of New Marker Genes Involved in Differentiation of Canine Adipose-Derived Stem Cells into Osteoblasts

**DOI:** 10.3390/ijms22136663

**Published:** 2021-06-22

**Authors:** Maurycy Jankowski, Mariusz Kaczmarek, Grzegorz Wąsiatycz, Claudia Dompe, Paul Mozdziak, Jędrzej M. Jaśkowski, Hanna Piotrowska-Kempisty, Bartosz Kempisty

**Affiliations:** 1 Department of Anatomy, Poznan University of Medical Sciences, 60-781 Poznan, Poland; mjankowski@ump.edu.pl; 2 Department of Cancer Immunology, Chair of Medical Biotechnology, Poznan University of Medical Sciences, 61-866 Poznan, Poland; markacz@ump.edu.pl; 3 Gene Therapy Laboratory, Department of Cancer Diagnostics and Immunology, Greater Poland Cancer Centre, 61-866 Poznan, Poland; 4 Department of Veterinary Surgery, Institute of Veterinary Medicine, Nicolaus Copernicus University in Torun, 87-100 Toruń, Poland; g.wasiatycz@umk.pl; 5 The School of Medicine, Medical Sciences and Nutrition, University of Aberdeen, Aberdeen AB25 2ZD, UK; claudia.dompe.16@abdn.ac.uk; 6 Prestage Department of Poultry Science, College of Agriculture and Life Sciences, North Carolina State University, Raleigh, NC 27695, USA; pemozdzi@ncsu.edu; 7 Department of Diagnostics and Clinical Sciences, Institute of Veterinary Medicine, Nicolaus Copernicus University in Torun, 87-100 Torun, Poland; jmjaskowski@umk.pl; 8 Department of Toxicology, Poznan University of Medical Sciences, 60-701 Poznan, Poland; hpiotrow@ump.edu.pl; 9 Department of Basic and Preclinical Sciences, Institute of Veterinary Medicine, Nicolaus Copernicus University in Toruń, 87-100 Torun, Poland; 10 Department of Histology and Embryology, Poznan University of Medical Sciences, 60-781 Poznan, Poland

**Keywords:** adipose, stem cells, RNAseq, transcriptome, analysis

## Abstract

Next-generation sequencing (RNAseq) analysis of gene expression changes during the long-term in vitro culture and osteogenic differentiation of ASCs remains to be important, as the analysis provides important clues toward employing stem cells as a therapeutic intervention. In this study, the cells were isolated from adipose tissue obtained during routine surgical procedures and subjected to 14-day in vitro culture and differentiation. The mRNA transcript levels were evaluated using the Illumina platform, resulting in the detection of 19,856 gene transcripts. The most differentially expressed genes (fold change >|2|, adjusted *p* value < 0.05), between day 1, day 14 and differentiated cell cultures were extracted and subjected to bioinformatical analysis based on the R programming language. The results of this study provide molecular insight into the processes that occur during long-term in vitro culture and osteogenic differentiation of ASCs, allowing the re-evaluation of the roles of some genes in MSC progression towards a range of lineages. The results improve the knowledge of the molecular mechanisms associated with long-term in vitro culture and differentiation of ASCs, as well as providing a point of reference for potential in vivo and clinical studies regarding these cells’ application in regenerative medicine.

## 1. Introduction

It is well known that the advancement of modern medicine will be highly dependent on the development of knowledge regarding stem cells [1,2,3,4,5,6]. While some studies focus on embryonic or induced pluripotent stem cells, the limited knowledge of stem cell regulatory mechanisms, as well as the significant risks associated with their administration, effectively delays any therapeutic approaches based on these cell types [7,8]. Adult progenitor cells, such as those derived from the bone marrow, have been effectively used in treatment of hematopoiesis-associated diseases (e.g., leukemia) since the 1970s [9,10]. Adult stem cells present a range of advantages over their pluripotent counterparts, such as their ready availability in the adult organism, a narrowed range of differentiation lineages, limiting the possibility of malignant development after implantation, and the possibility of collection without major surgical procedures [11,12,13,14,15]. Furthermore, a significant amount of recent research, especially regarding the treatment of bone- and cartilage-related diseases, focuses on adipose-derived stem cells (ASCs) [1,16,17]. Due to the ease of their extraction during the liposuction procedure, the wide array of works describing the methods of their culture and differentiation and their significant osteogenic and chondrogenic potential, these cells have become one of the most commonly investigated stem cell types, with a number of clinicians attempting to introduce effective treatments based on their autologous transplantation [18,19]. However, the exact molecular mechanisms governing the physiological and non-physiological functions of ASCs during their collection, long-term ex vivo culture, cryogenic storage or in vitro differentiation are still not entirely known [18]. Therefore, to enable the widespread adoption of this cell type in routine medical practice, a significant amount of wide-scale studies of the processes occurring during the procedures associated with current and potential applications of these cells are still needed to fully discover their actual clinical usefulness, as well as the possible risks associated with their grafting. The canine (*Canis familiaris*) source of stem cells is a relatively widely available model for human studies while also allowing for understanding canine stem cells as a potentially effective treatment in veterinary medicine [4,20,21].

Hence, the present study focuses on next-generation sequencing (RNAseq) analysis of genes with the biggest changes in their expression profile during long-term in vitro culture and osteogenic differentiation of canine adipose-derived stem cells (cASCs). The cASCs, isolated during routine surgical procedures, were subjected to culture and differentiation conditions that are, or that could be in the future, employed in procedures of in vitro expansion and induction of those cells towards specific lineages prior to autologous grafting. Hence, this study aims to analyze the genes with the most altered expression in the course of these processes, in order to identify new markers associated with the influence of the in vitro environment and induced osteogenic differentiation on this type of cells. This knowledge should allow better understanding the molecular mechanisms underlying the processes of ASC preparation for clinical application in veterinary and, potentially, human regenerative medicine.

## 2. Results

### 2.1. Flow Cytometry Analysis

**Figure 1:**
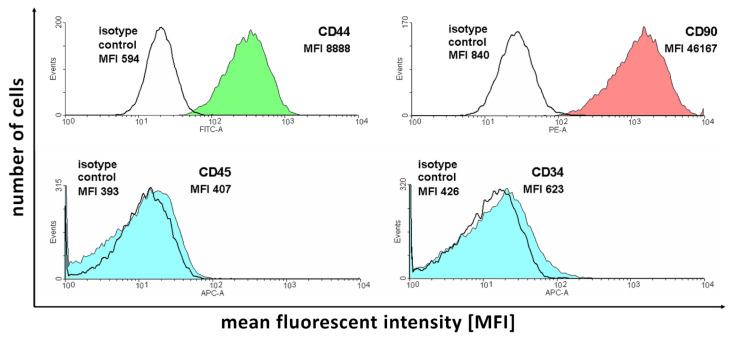
The results of flow cytometry analysis of selected ASC markers in the cell samples subjected to in vitro culture.

Flow cytometry analysis allowed evaluating the expression of the selected available canine ASC markers in the cells of interest to initially confirm their ability before further analysis (Figure 1).

Figure 1 presents the number of acquisition events for fluorescence signals of different intensities. Each of the analyzed antibodies was paired with the corresponding isotype control to correct the obtained results for background fluorescence unassociated with antibody binding. As it is presented in the figure, the two selected positive ASC markers (CD44 and CD90) present a large amount of detection events of larger fluorescence compared to the isotype controls. In turn, negative marker (CD45 and CD34) detection results show that the isotype control and antibody-stained samples show similar levels of fluorescence, confirming the absence of expression of these proteins in the analyzed cells. Overall, the results support the ASC identity of the isolated cell samples.

### 2.2. Morphological Evaluation and Osteogenic Differentiation

**Figure 2:**
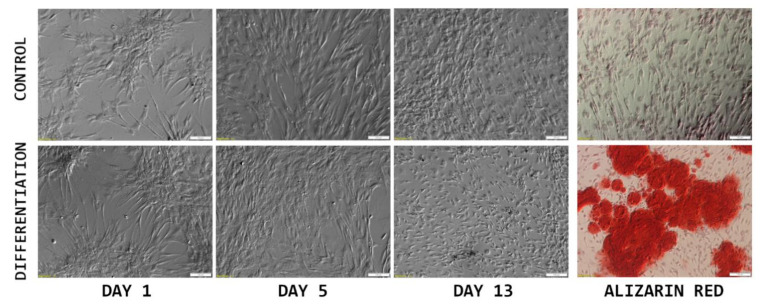
The results of morphological analysis of cASC primary culture (monochrome), combined with Alizarin Red staining photographs (color), to confirm osteogenic differentiation of the cells of interest. Scale bar: 100 μm.

As it is presented in Figure 2, the initially seeded cells exhibited a fibroblast-like elongated shape, typical for ASC cultures. After several days in culture (day 5), the cells presented a flattened, epithelial-like morphology, resulting from the increase in confluency. On day 13, at the end of the culture period, significant differences between the control and differentiated cells can be observed. While the control sample presents densely packed cells with a morphology typical for late cultures of >90% confluency, differentiated cells are small and more sparsely connected. After Alizarin Red staining, intense red coloring could be observed in the differentiated samples, while controls did not exhibit any color change.

### 2.3. RNAseq Analysis

**Figure 3:**
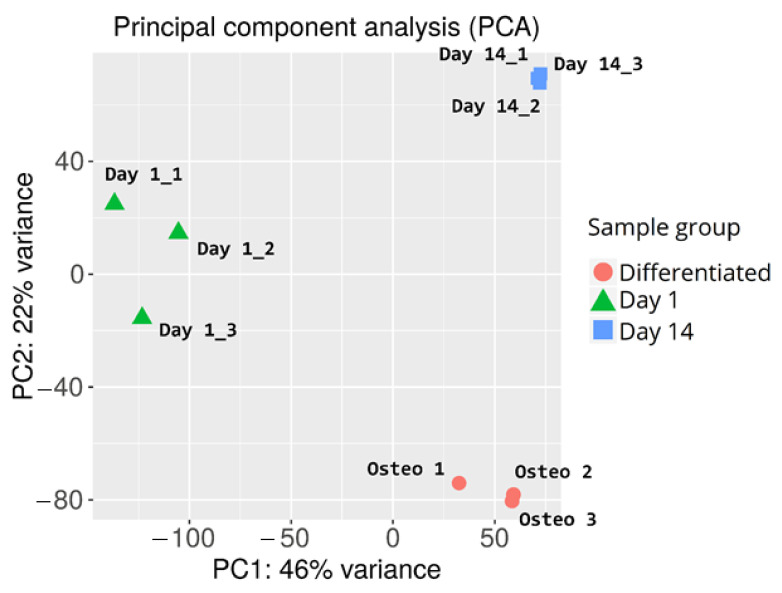
Principal component analysis of cASC samples subjected to RNAseq analysis.

During the initial analysis of the RNAseq results, principal component analysis of the samples was performed to examine variance between the analyzed sample groups and individual samples. The results of the PCA are presented in Figure 3.

Overall, there is a major variance between the sample groups, with less differences among samples belonging to the same group. This confirms the success of the differentiation procedure, as well as indicating similar gene expression changes evoked by long-term in vitro cultures of individual samples. The in-group variance can be observed in the day 1 control sample group, possibly due to the differences between the canine specimen from which the samples were harvested (e.g., age, condition, administered medication).

**Figure 4:**
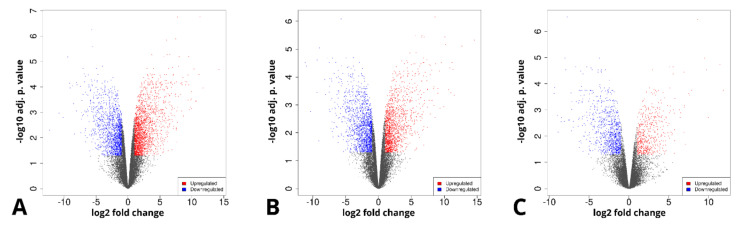
Volcano plots representing the composition of the analyzed sample groups, as well as the proportion and distribution of differentially expressed genes. (**A**)—day 14 vs. day 1, (**B**)—differentiated osteoblast vs. day 1, (**C**)—differentiated osteoblast vs. day 14.

Furthermore, genes that were differentially expressed between the analyzed sample groups were selected, based on a fold change of > |2| and an adj. *p* value of < 0.05. The initial results of this selection are presented in the form of volcano plots and compiled in Figure 4.

**Figure 5:**
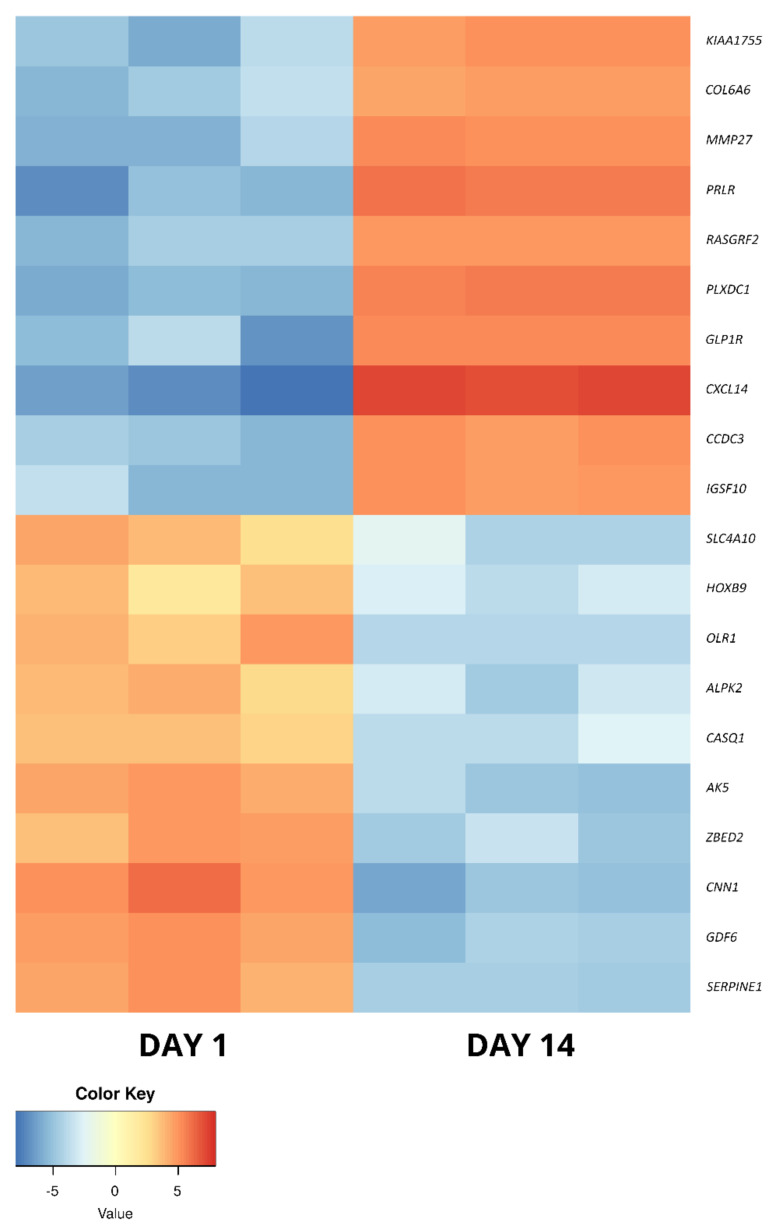
The heatmap representing the changes in the 10 most up- and downregulated genes between day 1 and day 14 of primary cASC culture, presented as log2FC.

**Figure 6:**
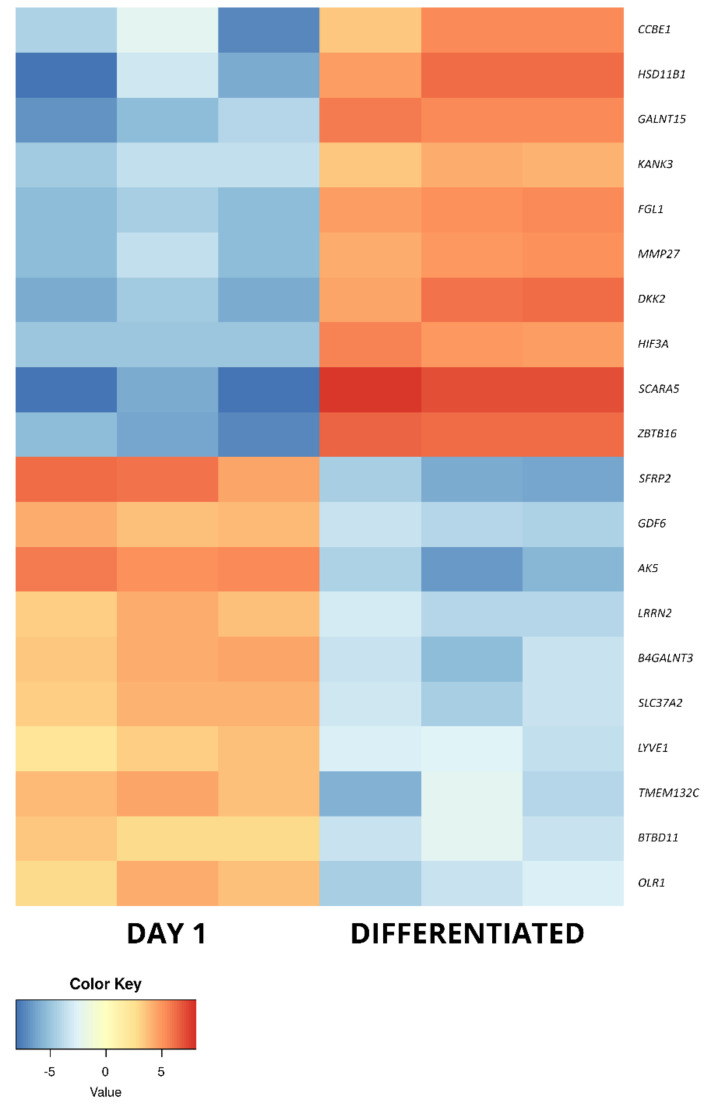
The heatmap representing the changes in the 10 most up- and downregulated genes between day 1 of primary cASC culture and differentiated osteoblasts, presented as log2FC.

**Figure 7:**
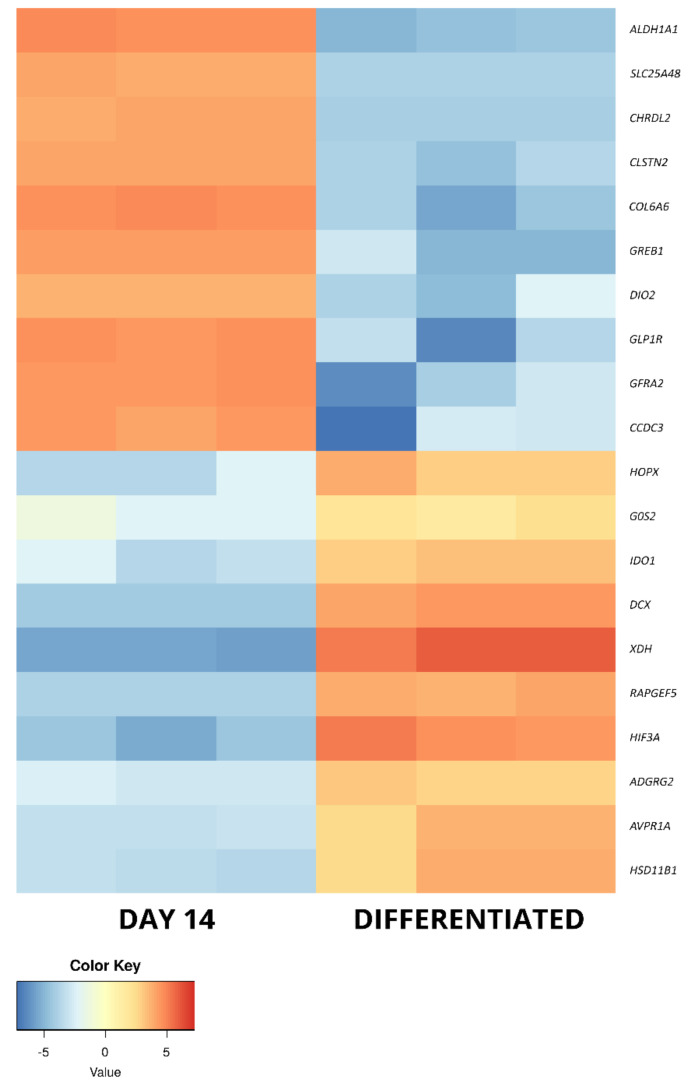
The heatmap representing the changes in the 10 most up- and downregulated genes between day 14 of primary cASC culture and differentiated osteoblasts, presented as log2FC.

The long-term in vitro culture of ASCs (day 14 vs. day 1) resulted in differential regulation of 3528 genes (1998 upregulated and 1536 downregulated), out of the total of 19,586 genes detected during the analysis (out of which 13,539 were successfully annotated with an Ensembl gene ID). In turn, similar parameters after osteogenic differentiation were presented by 3087 genes in relation to day 1 (1517 upregulated, 1570 downregulated) and by 1484 genes in relation to day 14 (548 upregulated and 963 downregulated). Out of these three gene sets, the 10 most up- and downregulated genes were selected for further analysis and are presented in the form of log2FC heatmaps (Figure 5, Figure 6 and Figure 7) and compiled in Table 1, together with their particular fold changes and adjusted *p* values.

Furthermore, some genes were differentially expressed between multiple analyzed sample groups (Table 2).

**Figure 8:**
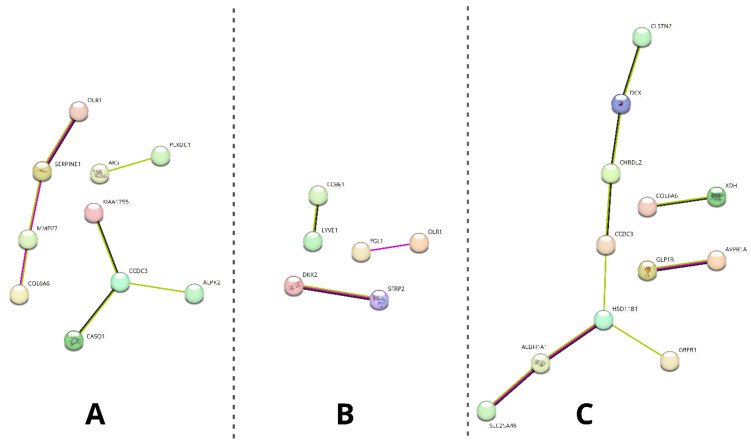
Results of the STRING analysis of predicted interactions between protein products encoded by the differentially analyzed genes of interest. The genes not involved in any interactions were excluded from the figure. (**A**)—day 14 vs. day 1, (**B**)—differentiated osteoblast vs. day 1, (**C**)—differentiated osteoblast vs. day 14. Colors of the edges indicate the source of predicted interaction: magenta—experimentally determined, green—textmining, black—co-expression.

Moreover, the differentially expressed gene of interest sets were uploaded to the STRING database to analyze their predicted interactions. The results of this analysis are presented in Figure 8.

As it is presented, different extents of predicted interactions were indicated among the analyzed sample groups (the highest in group C and the lowest in group B). Furthermore, a varying number of genes in each group were excluded from the analysis due to having no predicted interactions (10/20 on day 14 vs. day 1; 14/20 in differentiated osteoblasts vs. day 1; 8/12 in differentiated osteoblasts vs. day 14).

### 2.4. RT-qPCR Validation

**Figure 9:**
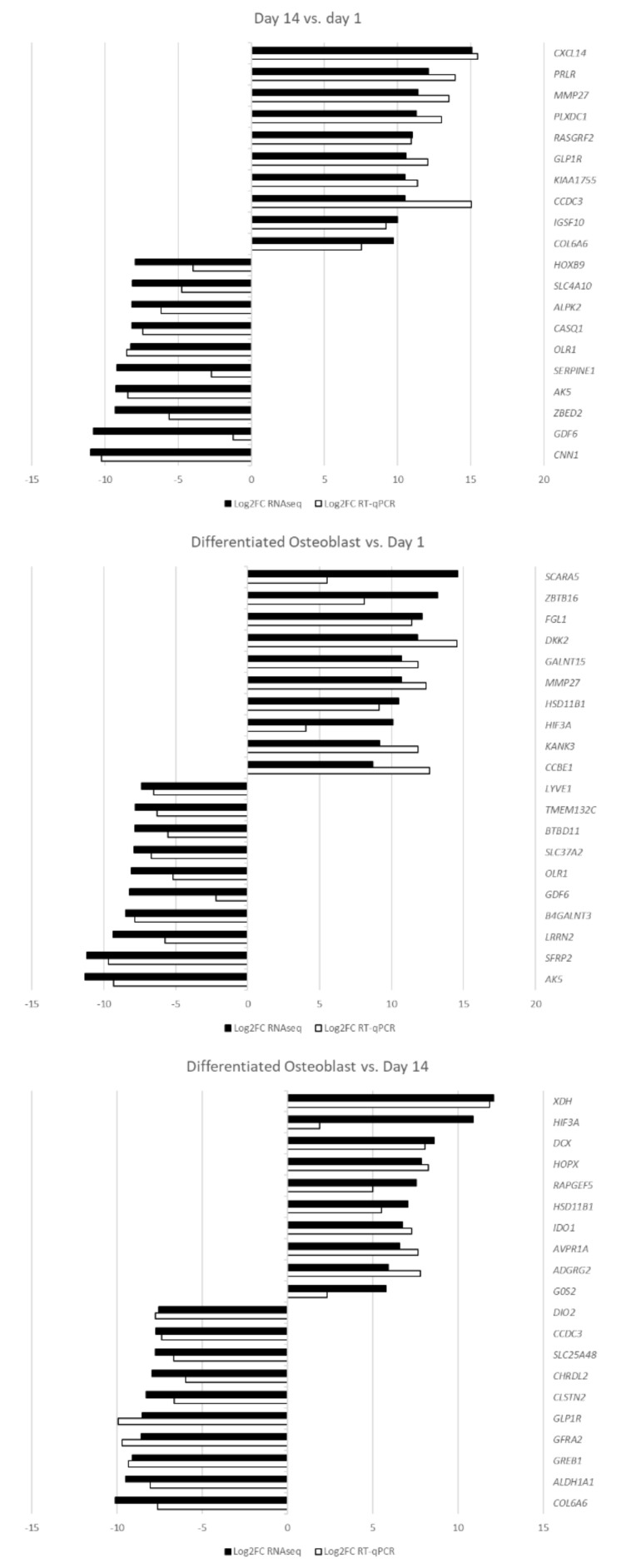
The results of RT-qPCR validation analysis presented as log2FC.

The sample transcript levels of the differentially expressed genes were validated by RT-qPCR using specific primers. The calculated log2FC results are presented as a bar graph in Figure 9.

The RT-qPCR analysis confirmed the direction of expression change in all of the samples. For most of the genes of interest, both methods showed comparable results, confirming the accuracy of the RNAseq analysis. However, some of the genes, namely, *GDF6*, *HIF3A*, *SCARA5* and *SERPINE1*, showed major differences in the extent of differential expression, most likely due to the differences in accuracy and sensitivity of the methods used. Nonetheless, all of the analyzed genes were validated as differentially expressed at FC ≥|2| and an adj. *p* value of < 0.05.

## 3. Discussion

In the presented study, RNAseq analysis was used to determine the transcriptomic changes associated with long-term in vitro cultures of ASCs, as well as their induced osteogenic differentiation. In the present day, next-generation sequencing (NGS) methods, such as RNAseq, allowed deeply investigating the molecular basis of various cell- and tissue-associated processes, especially when it comes to in vitro cultured cells [22]. This is especially important as adult stem cells, especially MSCs, have a significant potential to be differentiated into cells such as osteoblasts, chondrocytes and neuronal-like cells, which makes them particularly desirable in the context of use in regenerative and reconstructive medicine [11]. Complex transcriptomic analysis of cells with potential application in a clinical setting (e.g., ASCs) permits understanding the mechanisms caused by the ex vivo environment to which they are subjected during preparation for their administration [19]. Furthermore, some studies suggest that stem cells subjected to in vitro conditions have the potential for a significant increase in plasticity and even potential differentiation towards lineages unassociated with the tissue of origin [23]. Moreover, there is an increasingly prevalent discussion regarding the potential differentiation of stem cells prior to application, allowing for ensuring their proper lineage progression and minimizing the risk of malignant progression upon patient administration [19,24]. However, a lot of doubt remains about whether such approach would be beneficial for the clinical results [13]. Hence, the NGS approach can be employed to obtain extensive data on the variation in gene expression observed during the usual procedures conducted during clinical administration of stem cells, and to evaluate the process of the included differentiation for future medicinal use [25]. Furthermore, bioinformatical analysis of the results allows identifying the most differentially expressed genes during such processes that could serve as their reference markers of the effects of in vitro culture and induced differentiation on ASCs, for both future follow-up studies and potential clinical evaluation [26].

Firstly, day 14 samples were compared to those collected at day 1 to show the molecular effects of ASC long-term in vitro culture, suggested as a way to increase their concentration and eliminate contamination with other cell types for clinical application [19]. Among the 10 most upregulated genes between these sample groups, eight were previously associated with stem cell-related processes. When it comes to genes showing elevated expression after 14 days of culture, both *CXCL14* and *PRLR* have been described to take part in the general processes of mesenchymal stem cell (MSC) proliferation and differentiation [27,28]. Similar functions have been attributed to *GLP1R* and *CCDC3*, with their participation in the processes of osteogenic and adipogenic differentiation [29,30,31]. Furthermore, the latter gene’s expression was also observed during ASC immortalization, with a possible implication in cancer progression [32]. *PLXDC1* has also been widely described in the context of malignancy, especially metastasis, and has been previously detected during analysis of mesenchymal stem cells [33]. In turn, *RASGRF2* was also detected in previous MSC analyses, particularly in cells derived from the umbilical cord [34]. Moreover, it has been found to play a role in the regulation of stem cell density and neural differentiation during adult neurogenesis [35]. The next gene, IGSF10, has been previously detected to be upregulated during osteoblast differentiation, with an implied role in the ossification process [36]. Finally, *COL6A6* has been implicated in the process of vascular smooth muscle cell differentiation from pluripotent progenitors [37]. The remaining most upregulated genes in this comparison, not previously described in the context of stem cells, were *MMP27* and *KIAA1755*. The former encodes a matrix metalloproteinase particularly involved in stromal breakdown, which was connected to the product of *COL6A6* in the STRING analysis [38]. *KIAA1755* has, only as of now, been implied to have a function in the cardiac rhythm and rate, which does not shed further light on its potential role during long-term in vitro culture of ASCs [39]. However, it is suggested that it has a role in the culture-induced differentiation, as the STRING analysis showed a potential interaction of its protein product with that of the previously described *CCDC3*.

The most downregulated genes are all associated with stem cell differentiation. *HOXB9*, a member of the highly conserved and multifunctional homeobox genes’ group, was implied in the normal functioning of stem cells [40]. Its abnormal expression, particularly upregulation, was detected in, e.g., colorectal cancer cells [41]. Similar roles were attributed to *GDF6* and *CNN1*, as they were implied in MSC and adipocyte lineage commitment and contractile MSC differentiation, respectively [42,43]. Furthermore, *AK5*, *ALPK2* and *CASQ1* are all associated with pluripotent stem cells. The first of those genes, *AK5*, was indicated as a marker of cells derived from embryoid bodies [44]. In turn, *ALPK2* and *CASQ1* were detected in pluripotent stem cell-derived lineages. The expression of the former was noted in cardiac cell development, while the latter was implicated in neurogenic differentiation [45,46,47]. The presence of products of two further genes, *SLC4A10* and *SERPINE1*, was detected in the content of mesenchymal stem cell-derived exosomes [48,49]. In turn, *OLR1* encodes a proinflammatory protein that is often observed at low levels in ASCs, with elevated expression in cells obtained from diabetic and obese animals, predisposing them to an adipogenic fate [50]. Finally, *ZBED2* has been implicated in the process of keratinocyte differentiation, with its depletion resulting in a basal phenotype of increased plasticity [51].

In this comparison, a large number of genes participating in the processes of MSC differentiation were majorly upregulated between day 14 and day 1 of primary culture. This fact, together with the downregulation of several genes responsible for plasticity, suggests the potential of ASCs for lineage commitment after long-term in vitro cultures.

In the current study, the most differentially regulated genes after osteogenic differentiation of ASCs were also investigated. The differentiated osteoblasts were compared to the day 1 control cells, as well as those cultured in vitro for 14 days, in an attempt to correct for the expression changes induced solely by long-term culture. When compared to day 1, 4 out of 10 upregulated genes were associated with an osteoblast phenotype. *ZBTB16*, *DKK2* and *HSD11B1* were previously described to take part in osteogenic differentiation, with the last gene of this set also detected in osteoblasts in vivo [52,53,54,55,56]. In turn, *SCARA5* activity was previously reported to prevent osteoblast differentiation, suggesting a potential inhibiting response of ASCs to the inducing medium [57]. Four further genes were associated with an MSC-related process but were previously implicated only in the differentiation towards other mesenchymal lineages. *FGL1* was found to be upregulated in dental pulp stem cells, as compared to osteoblasts [58]. In turn, *GALNT15* was described as upregulated during adipocyte differentiation, while HIF3A and *CCBE1* were, respectively, associated with chondrocyte and cardiac phenotypes [59,60,61]. Among the last two of the analyzed most upregulated genes between differentiated osteoblasts and day 1 ASC culture, *KANK3* was linked to cancer processes as a tumor suppressor gene, while *MMP27* was already detected to be upregulated after long-term in vitro culture, suggesting its overexpression is not directly connected to the differentiation process [62,63].

Among the most downregulated genes in osteoblasts vs. day 1 of culture, 9 out of 10 were previously described in the context of stem cells. Among them, three (*OLR1*, *GDF6* and *AK5*) presented a similar downregulation after long-term in vitro culture, making it unlikely that they are directly linked to the process of differentiation. Furthermore, four further genes were found to be expressed in MSCs. *BTBD11* was detected in MSCs derived from the umbilical cord, while *SLC37A2* is expressed by bone marrow- and hematopoietic stem cell-derived lineages [34,64]. In turn, *B4GALNT3* was found to be upregulated in 3D cultures of MSCs [65]. While *SFRP2* is certainly connected to the mesenchymal differentiation potential, its exact roles seem to be conflicting, with sources reporting its involvement in adipogenic, neurogenic and osteogenic progression ability [66,67]. *LYVE1* has also been implicated in differentiation processes, marking lymphatic-like phenotype transformation in vitro [68]. Finally, while *NLRR2* was noted to be a differentiation suppressor, the only studies reporting on this gene come from research focused on malignant tumor cells [69]. The last gene in this set, *TMEM132C*, is not yet well described in the literature [70]. However, its downregulation after osteogenic differentiation of ASCs might suggest it as a marker of their stemness and/or differentiation into lineages other than osteogenic.

An upregulation of osteoblast markers was evident, coupled with a downregulation in the expression of genes characteristic for other MSC types. However, as some of the most upregulated genes were previously associated with other mesenchymal lineages, their role in the process of in vitro osteogenic differentiation should be reconsidered. Furthermore, significant upregulation of SCARA5 indicates a previously unseen response of ASCs to induce osteogenic differentiation.

Among the genes that were upregulated between differentiated osteoblasts and day 14 of in vitro culture, two (*HSD11B1* and *HIF3A*) were also upregulated in relation to day 1. This further supports the notion that they are important in the processes of osteogenic differentiation and not just related to the influence of long-term in vitro conditions. Furthermore, there are three more genes in this set that have been previously associated with osteoblast differentiation. *HOPX* was described to drive osteogenesis through adipogenesis suppression, while *IDO1* is involved in the kynurenine pathway activated during the osteoblast differentiation process [71,72]. In turn, *RAPGEF5* is a major regulator of nuclear translocation of β-catenin, a mechanism important in canonical Wnt signaling pathways that play a significant role in osteogenic differentiation [73]. Among the next two genes associated with stem cells, *G0S* was found to maintain quiescence of hematopoietic progenitor cells, while *DCX* is a known marker of central nervous system progenitor cells [74,75]. Furthermore, both *XDH* (downregulated in liver tumors) and *AVPR1A* (upregulated in prostate cancer) have been implicated in the activity of cancer-related stem cells [76,77]. The final gene of this set, *ADGRG2*, does not seem to have any association with stem cell-related processes, only being described in recent studies as a marker of male fertility [78]. However, STRING analysis indicated its potential interaction with *GLP1R*, suggesting its possible undescribed participation in the processes associated with long-term in vitro cultures of ASCs.

The final set of the most downregulated genes between osteoblasts and day 14 culture cells contains three genes that were previously detected to be upregulated during the day 14 vs. day 1 comparison (*CCDC3*, *GLP1R*, *COL6A6*). This result suggests that an elevated expression of these genes is only associated with long-term in vitro culture of ASCs and does not play a role during their osteogenic differentiation. Three further genes, *DIO2*, *CLSTN2* and *GREB1*, were previously described to be upregulated in osteoblasts compared to MSCs. However, the obtained results suggest that their change in expression is rather a result of long-term in vitro culture conditions than osteogenic induction of ASCs [79,80,81]. The next two genes of this set, *CHRDL2* and *GFRA2*, are involved in differentiation of chondrocytes and cardiomyocytes, respectively [82,83]. In turn, *ALDH1A1* is a known cancer stem cell marker [84]. The last gene analyzed in this study during this analysis was *SLC25A48*, a member of the *SLC25* mitochondrial transporter protein without a described function in the context of stem cells [85]. However, it is worth noting that it has shown a predicted interaction with *ALDH1A1* in the STRING analysis.

In conclusion, upregulation of several osteogenesis-related genes between differentiated osteoblasts and day 14 of primary culture further confirms the success of the differentiation process, as well as providing further proof of the role of these genes as osteogenic differentiation markers. Furthermore, an expected downregulation of genes related to other differentiation lineages was observed, together with a few genes previously reported to be upregulated in comparison to undifferentiated MSCs, suggesting that some of these genes could be upregulated due to the influence of in vitro culture conditions rather than induced osteoblast differentiation. Overall, this study provides further molecular insight into the processes that occur during long-term in vitro culture and osteogenic differentiation of ASCs. The results allow confirming currently known markers of osteogenic differentiation (e.g., *HSD11B1*, *ZBTB16* and *DKK2*), re-evaluating the roles of some genes in MSC progression towards a range of lineages (e.g., *GALNT15*, *HIF3A* and *CCBE1*) and suggesting new markers associated with the influence of in vitro conditions and induced differentiation on these cells (e.g., *CCDC3*, *GLP1R* and *COL6A6*). Finally, some genes of currently unknown function, such as *KIAA1755* and *TMEM132C*, were found to be among the most regulated during the analyzed processes of ASCs in in vitro culture and osteoblast differentiation, providing a reference for further studies of their potential role in regenerative and reconstructive medicine as well as translational research.

## 4. Materials and Methods

### 4.1. Material Collection

The research material, in the form of small (<1 cm^3^) samples of adipose tissue, was obtained from a young c. familiaris specimen subjected to a routine sterilization procedure at an associated veterinary clinic. The material analyzed in this study is usually discarded after the surgery. Hence, it does not require any additional surgical procedures. Therefore, this study was deemed to be exempt from approval of the local bioethical committee. After collection, the samples were placed in Dulbecco’s phosphate-buffered saline (DPBS; Sigma-Aldrich, Saint Louis, MO, USA), supplemented with 1% of antibiotic antimycotic solution (A5955, Sigma-Aldrich, Saint Louis, MO, USA). The material was then stored at 4 °C and transported to the laboratory no longer than 24 h after collection.

### 4.2. Cell Sample Preparation

The adipose tissue samples were first double washed with ice-cold PBS to remove any remnant blood. Furthermore, they were minced in a Petri dish using sterile surgical blades until homogenous. After such preparation, the samples were subjected to enzymatic digestion in a 1 mg/mL solution of Type I collagenase (Gibco, Thermo-Fischer Scientific, Waltham, MA, USA) for 40 min at 37 °C, with vortexing every 10 min of incubation. Afterwards, the enzyme activity was stopped with addition of 1 mL of fetal bovine serum (FBS, Sigma-Aldrich, Saint Louis, MO, USA), and the sample was centrifuged at 1200× *g* for 10 min. Supernatant liquid was collected from above the resulting cell pellet, which was resuspended in DPBS and centrifuged again at 500× *g* for 10 min. After discarding the supernatant, the obtained cells were resuspended in 4 mL of Dulbecco’s Modified Eagle’s Medium—high glucose (DMEM, Sigma-Aldrich, Saint Louis, MO, USA), supplemented with 10% FBS (Sigma-Aldrich, Saint Louis, MO, USA), 4 mM of L-glutamine (Sigma-Aldrich, Saint Louis, MO, USA) and 1× antibiotic-antimycotic solution (A5955, Sigma-Aldrich, Saint Louis, MO, USA). The resulting suspension was transferred into 25 cm^3^ cell culture flasks (two flasks for each canine specimen, in order to ensure the supply of early controls without additional disturbance of differentiation samples).

### 4.3. Flow Cytometry Analysis

At the beginning of cell culture, a sample of cell-containing medium was collected for antibody staining and flow cytometry analysis. The cells were stained with the following antibodies: rat anti-dog CD44: FITC (11-5440-41, Thermo-Fischer Scientific, Waltham, MA, USA), rat anti-dog CD90: PE (12-5900-41, Thermo-Fischer Scientific, Waltham, MA, USA), rat anti-dog CD45: APC (MCA1042APC, Bio-Rad, Hercules, CA, USA) and mouse anti-dog CD34: Alexa Fluor^®^ 647 (MCA2411A647, Bio-Rad, Hercules, CA, USA), as well as respective isotype controls: rat IgG2ak: FITC (11-5440-41, Thermo-Fischer Scientific, Waltham, MA, USA), rat IgG2bk: PE (12-4031-81, Thermo-Fischer Scientific, Waltham, MA, USA), rat IgG2b: APC (MCA6006APC, Bio-Rad, Hercules, CA, USA) and mouse IgG1: Alexa Fluor^®^ 647 (MCA928A647, Bio-Rad, Hercules, CA, USA). All of the antibodies and isotype controls were applied at 1:50 dilution, with staining performed according to manufacturer protocols. Stained samples were subjected to flow cytometry using the BD FACSAria™ cytometer (Becton Dickinson, Franklin Lanes, NJ, USA).

### 4.4. In Vitro Cell Culture

The initial in vitro cultures (IVC) were conducted for a period of 3 days, or until ~90% confluency was observed, with the exception of early control samples that were harvested for RNA isolation after 1 day of culture. Afterwards, the cells were detached from culture bottles using 1× Trypsin solution (Sigma-Aldrich, Saint Louis, MO, USA) and passaged onto 6-well culture plates. There, they were maintained in DMEM until the 14th day of culture, with medium changed every 72 h. Furthermore, photographs of the cultures were taken, using an inverted microscope and proprietary software (Ixplore Standard, Olympus, Tokyo, Japan), every 24 h to assess morphological changes of the analyzed cells.

### 4.5. Osteogenic Differentiation

Three out of six wells of each 6-well culture plate were subjected to osteogenic differentiation. For that purpose, after the initial adherence period, DMEM was replaced with canine osteogenic differentiation medium (Cn417D, Cell Applications Inc., San Diego, CA, USA), supplemented with 1% of antibiotic-antimycotic solution (A5955, Sigma-Aldrich, Saint Louis, MO, USA). The culture was then maintained for a period of 14 days, with medium changed every 72 h. Photographs of the samples were taken every 24 h to monitor the progression of culture. After this period, sample control and differentiated cultures were fixed in Saccomanno solution (Morphisto GmbH, Offenbach am Main, Germany) and stained with Alizarin Red (A5533, Sigma-Aldrich, Saint Louis, MO, USA), according to the manufacturer protocols, in order to ensure the success of osteogenic differentiation. Each 6-well culture plate consisted of material collected from a separate canine specimen. One well out of each technical triplicate was stained with Alizarin Red. The resulting staining and morphological differences of control and differentiated samples were visually confirmed using an optical microscope and proprietary software (Ixplore Standard, Olympus, Tokyo, Japan) and the differentiation medium manufacturer’s visual guidelines, in order to ensure the success of the differentiation process. Furthermore, the two remaining culture wells of both control and differentiated samples were pooled together, representing a single set of biological samples.

### 4.6. RNA Isolation

RNA samples were isolated at three time periods of ASC culture: 1 day of culture (early control), 14 days of culture in DMEM (long-term IVC) and 14 days of culture in osteogenic differentiation media (differentiated osteoblasts). The cells directed for RNA isolation were detached from cultures using 1× Trypsin solution and placed in 1mL of TRIzol (Thermo-Fischer Scientific, Waltham, MA, USA). The samples were then frozen at −80°C. The isolation of RNA was conducted using the TRIzol Plus Purification KIT (12183555, Thermo-Fischer Scientific, Waltham, MA, USA) according to the manufacturer’s protocol. The total amount of the collected RNA was then evaluated based on optical density at 260 nm, with its purity determined using the 260/280 nm absorption ratio (NanoDrop 2000 spectrophotometer, Thermo-Fischer Scientific, Waltham, MA, USA). Only samples with RNA content over 1 mg and a 260/280 absorption ratio higher than 1.8 were subjected to further studies.

### 4.7. RNAseq Analysis

The RNAseq analysis of the samples was performed at CeGaT GmbH (Tübingen, Germany) using the Illumina platform (Illumina, San Diego, CA, USA). The samples consisted of three biological repeats for each of the analyzed culture periods, with three sets of day 1, day 14 and osteogenic samples obtained from three different animals, in order to correct for inter-specimen differences. Before the analysis, additional quality control of the samples was performed using Qubit RNA (Thermo-Fischer Scientific, Waltham, MA, USA) and Bioanalyzer RNA (Aglient Technologies, Santa Clara, CA, USA). RNA integrity numbers (RINs) of the samples were determined at values between 9.3 and 10, allowing for further processing. The cDNA library used for the subsequent analysis was prepared from 100 ng of RNA per sample using TruSeq Stranded mRNA kit (Illumina, San Diego, CA, USA). The sequencing was performed using NovaSeq 6000 (Illumina, San Diego, CA, USA). Afterwards, demultiplexing of the sequencing reads was performed using bcl2fastq (v. 2.20), with adapters trimmed with Skewer (v. 0.2.2) [86]. The trimmed raw reads were aligned to the CanFam3.1 canine genome using STAR (v. 2.5.2b).

### 4.8. RT-qPCR Validation

The RNA material remaining after RNAseq analysis was subjected to reverse transcription using Transcriptor First Strand cDNA Synthesis Kit (Roche Life Sciences, Basel, Switzerland) and the Eppendorf Mastercycler ^®^ nexus (Eppendorf AG, Hamburg, Germany), according to the manufacturer protocols. The RT-qPCR validation was then performed on a Lightcycler 96 (Roche Life Sciences, Basel, Switzerland) using Eva Green (Syngen Biotech, Wrocław, Poland) as a detection dye. The final reaction mix consisted of 1 μL of cDNA, 1 μL of forward+reverse primer mix, 2 μL of Eva Green and 6 μL of PCR-grade water. The specific primers were designed based on Ensembl transcript sequences [87], using the Primer3 software (Table 3) [88]. The design process was based on the exon–exon method to avoid potential remnant genomic DNA amplification. Furthermore, the primers were designed to match all of the known protein-coding transcript variants. The results of the analysis were calculated based on the 2-ΔΔCT method, with *ACTB* and *HPRT* used as housekeeping genes [89].

### 4.9. Bioinformatical and Statistical Analysis

The raw counts obtained from the initial analysis contained the numbers of reads assigned to particular geneIDs. These datasets were subjected to normalization using the DESeq2 package (v. 1.24) in R (v. 4.0.3). Normalized gene lists were then annotated with ENTREZ gene numbers using the org.cf.eg.db annotation package included in Bioconductor (v.3.12.0), in order to provide reference for further analyses. The obtained ENTREZ annotated datasets were uploaded to the IDEP.91 interface for further processing and visualization. IDEP is an integrated web application, providing a simple user interface for advanced bioinformatical analysis of RNAseq data [90].

Principle component analysis (PCA) was used to visualize the relationship between samples. For this purpose, EdgeR transformation was used to stabilize the variance across the mean, allowing all of the genes to equally contribute to the distance between samples [91].

Furthermore, differentially expressed gene (DEG) lists were extracted. For that purpose, fold change (FC) was based on the mean expression values of each gene in the three analyzed sample groups. Furthermore, statistical significance of the results was determined based on the p. value obtained from a Wald test, corrected for multiple comparisons with the use of Benjamini and Hochberg’s false discovery rate. The selection of DEGs was therefore based on FC ≥ 2 and adjusted *p* value ≤ 0.05. The results of the selection were presented as a volcano plot, on which each point signifies a gene, with the log2 of the fold change and adj. *p* value presented on the *y* and *x* axes, respectively. For the purpose of this study, the 10 most upregulated and downregulated genes between the sample groups (day 14 vs. day 1; differentiated vs. day 1; differentiated vs. day 14) were selected. The expression of the genes of interest was visualized in the form of heatmaps, with the intensity of each sample’s color indicating the extent of the change in expression between sample groups.

Finally, all of the gene of interest lists were uploaded to the STRING (Search Tool for Retrieval of Interacting Genes/Proteins, STRING Consortium, Lausanne, Switzerland) database to determine the predicted interaction between the analyzed genes. The STRING database contains data on protein/gene interactions that have been obtained through experimentation, computational forecasting methods and analyses of the available public literature [92].

## Figures and Tables

**Table 1:** The list of differentially expressed genes of interest analyzed in this study. FC—fold change; adj. *p* value—adjusted *p* value.

Day 14 vs. Day 1				
Gene Symbol	FC	Log2FC	adj. *p* Value	Entrez Gene ID
*CXCL14*	3.48 × 10^4^	15.1	9.64 × 10^−5^	610078
*PRLR*	4.53 × 10^3^	12.1	3.59 × 10^−4^	479363
*MMP27*	2.70 × 10^3^	11.4	1.31 × 10^−3^	489430
*PLXDC1*	2.53 × 10^3^	11.3	9.06 × 10^−6^	491032
*RASGRF2*	1.98 × 10^3^	11.0	7.71 × 10^−6^	610203
*GLP1R*	1.53 × 10^3^	10.6	9.98 × 10^−6^	481778
*KIAA1755*	1.45 × 10^3^	10.5	5.58 × 10^−4^	485868
*CCDC3*	1.42 × 10^3^	10.5	2.56 × 10^−4^	607593
*IGSF10*	1.03 × 10^3^	10.0	3.54 × 10^−3^	477114
*COL6A6*	8.57 × 10^2^	9.74	7.93 × 10^−6^	610649
*HOXB9*	4.14 × 10^−3^	−7.92	3.15 × 10^−2^	608958
*SLC4A10*	3.61 × 10^−3^	−8.11	4.24 × 10^−2^	478766
*ALPK2*	3.48 × 10^−3^	−8.17	2.47 × 10^−2^	483966
*CASQ1*	3.48 × 10^−3^	−8.17	2.00 × 10^−3^	608401
*OLR1*	3.30 × 10^−3^	−8.24	3.24 × 10^−2^	486694
*SERPINE1*	1.73 × 10^−3^	−9.17	2.19 × 10^−2^	403476
*AK5*	1.63 × 10^−3^	−9.26	2.18 × 10^−3^	490204
*ZBED2*	1.60 × 10^−3^	−9.29	1.19 × 10^−2^	487971
*GDF6*	5.47 × 10^−4^	−10.8	1.10 × 10^−3^	100686579
*CNN1*	4.89 × 10^−4^	−11.0	2.89 × 10^−2^	484937
**Differentiated Osteoblast vs. Day 1**				
Gene Symbol	FC	Log2FC	adj. *p*. value	Entrez Gene ID
*SCARA5*	2.55 × 10^4^	14.6	5.55 × 10^−3^	486097
*ZBTB16*	9.73 × 10^3^	13.2	2.04 × 10^−4^	489398
*FGL1*	4.39 × 10^3^	12.1	4.58 × 10^−3^	475617
*DKK2*	3.64 × 10^3^	11.8	4.36 × 10^−2^	478502
*GALNT15*	1.66 × 10^3^	10.7	1.32 × 10^−3^	477056
*MMP27*	1.65 × 10^3^	10.7	1.18 × 10^−2^	489430
*HSD11B1*	1.45 × 10^3^	10.5	2.40 × 10^−2^	449023
*HIF3A*	1.11 × 10^3^	10.1	6.36 × 10^−3^	476429
*KANK3*	5.85 × 10^2^	9.19	1.09 × 10^−2^	476723
*CCBE1*	4.09 × 10^2^	8.68	4.90 × 10^−2^	610092
*LYVE1*	6.03 × 10^−3^	−7.37	2.20 × 10^−2^	100855439
*TMEM132C*	4.44 × 10^−3^	−7.82	8.64 × 10^−3^	403528
*BTBD11*	4.34 × 10^−3^	−7.85	1.86 × 10^−3^	481292
*SLC37A2*	4.22 × 10^−3^	−7.89	4.85 × 10^−3^	489303
*OLR1*	3.64 × 10^−3^	−8.10	4.15 × 10^−2^	486694
*GDF6*	3.39 × 10^−3^	−8.20	1.86 × 10^−3^	100686579
*B4GALNT3*	2.73 × 10^−3^	−8.51	9.29 × 10^−3^	486751
*LRRN2*	1.52 × 10^−3^	−9.36	8.64 × 10^−3^	488564
*SFRP2*	4.25 × 10^−4^	−11.2	3.41 × 10^−2^	475471
*AK5*	3.90 × 10^−4^	−11.3	3.40 × 10^−3^	490204
**Differentiated osteoblast vs. Day 14**				
Gene Symbol	FC	Log2FC	adj. *p*. value	Entrez Gene ID
*XDH*	4.26 × 10^3^	12.1	3.61 × 10^−3^	483028
*HIF3A*	1.89 × 10^3^	10.9	6.74 × 10^−3^	476429
*DCX*	3.89 × 10^2^	8.60	8.85 × 10^−4^	612950
*HOPX*	2.31 × 10^2^	7.85	4.04 × 10^−2^	100855799
*RAPGEF5*	1.88 × 10^2^	7.56	2.47 × 10^−2^	100855786
*HSD11B1*	1.32 × 10^2^	7.05	3.15 × 10^−2^	449023
*IDO1*	1.08 × 10^2^	6.76	8.60 × 10^−3^	475574
*AVPR1A*	97	6.60	1.59 × 10^−2^	481142
*ADGRG2*	60.6	5.92	5.00 × 10^−4^	491763
*G0S2*	54.7	5.77	2.39 × 10^−2^	609704
*DIO2*	5.34 × 10^−3^	−7.55	2.37 × 10^−8^	490813
*CCDC3*	4.72 × 10^−3^	−7.73	3.49 × 10^−4^	607593
*SLC25A48*	4.65 × 10^−3^	−7.75	5.44 × 10^−5^	481518
*CHRDL2*	4.08 × 10^−3^	−7.94	1.97 × 10^−6^	609312
*CLSTN2*	3.17 × 10^−3^	−8.30	5.39 × 10^−6^	477093
*GLP1R*	2.74 × 10^−3^	−8.51	1.15 × 10^−5^	481778
*GFRA2*	2.58 × 10^−3^	−8.60	5.41 × 10^−5^	609172
*GREB1*	1.84 × 10^−3^	−9.08	9.19 × 10^−5^	610007
*ALDH1A1*	1.38 × 10^−3^	−9.50	9.19 × 10^−5^	476323
*COL6A6*	8.90 × 10^−4^	−10.1	9.68 × 10^−6^	610649

**Table 2:** The list of genes that exhibited differential expression in multiple comparisons between sample groups, with the direction and extent of expression change compared in the form of log2 fold change.

	Log2FC		
	Day 14 vs. Day 1	Osteoblast vs. Day 1	Osteoblast vs. Day 14
*AK5*	−9.26	−11.30	
*CCDC3*	1.05		−7.73
*COL6A6*	9.74		−10.10
*GDF6*	−10.80	−8.20	
*GLP1R*	10.60		−8.51
*HIF3A*		10.10	10.90
*HSD11B1*		10.50	7.05
*MMP27*	11.40	10.71	
*OLR1*	−8.24	−8.10	

**Table 3:** The sequences of primers used in this study, with Ensembl IDs of the genes used for their design included.

Gene	Ensembl ID	Forward	Reverse
*XDH*	ENSCAFG00000005609	AGACTGCTCACGGGTCATTC	ACTCGCTCGCCTTTAAAACA
*HIF3A*	ENSCAFG00000045412	AGTGGTGGCTATCTGTGGAC	AGAGTGTCTGCGAGAGTGTC
*DCX*	ENSCAFG00000018179	CGGAAGCATGGATGAACTGG	GTCCTTGTTCTCTCTGGCCT
*HOPX*	ENSCAFG00000031330	TCAGCAAGGTCAACAAGCAC	TCACGGATCTGCACTCTGAG
*RAPGEF5*	ENSCAFG00000002731	ATGATGAAGTGGCGACCGTT	CAATCACTCTCGGCGCTG
*HSD11B1*	ENSCAFG00000011925	CTTCTCCACTTGTTGCACCC	TCCAGGGCACATTCTTCCTT
*IDO1*	ENSCAFG00000005750	AAACATGTGGACCCAAGCAC	CAGCAGCGAATTCTTCACCA
*AVPR1A*	ENSCAFG00000000339	CAGCAGCGTGAAGACCATTT	GGTGATGGCTGGGTTTTCTG
*ADGRG2*	ENSCAFG00000012983	ATGGCCTTGGATCCTATGGG	AACGCTCCTGAGGTCTTGAA
*G0S2*	ENSCAFG00000030095	CTGGTGAAGCTGTACGTGC	ATCACCACGCCCAGGAAG
*DIO2*	ENSCAFG00000032243	TCTCCAACTGCCTCTTCCTG	CTTCACCCAGTTTCACCTGC
*CCDC3*	ENSCAFG00000030997	ACCCTGGAGAAGCGTAACAA	CCAGGGCATTAATGTGTGGG
*SLC25A48*	ENSCAFG00000001085	GTTTGGCTTCTTCAAGGGCA	CAGGAGAAGGTCAGACAGGG
*CHRDL2*	ENSCAFG00000005475	CTCCAAGCCCAGACAACCTA	GGTTGCTTTGTCTGGACTGG
*CLSTN2*	ENSCAFG00000007635	CATTCAAGATCCACGGCCAG	ACTTCTTCCAGGCTGTCTCC
*GFRA2*	ENSCAFG00000010049	CATCTCCATCTGCAACCGTG	GGCTTCTCCTTGTCCTCGTA
*GREB1*	ENSCAFG00000003579	GAGCACATGACGAAGCAGAG	TCCTTCACCTCCCTGCAATT
*ALDH1A1*	ENSCAFG00000001791	AGGAGTGTTGAACGGGCTAA	TTTGAGAAAACGGTGGGCTG
*SCARA5*	ENSCAFG00000008354	CGATGAGGGGAAGATGGGAG	CCCTGCTCTGTCTCCTTTGT
*ZBTB16*	ENSCAFG00000013538	GATGACAATGACACGGAGGC	CATGGCTGAAGGACCGAATG
*FGL1*	ENSCAFG00000032313	AAAACAGCCGCTATGCACAA	CACCTGTTAAACCACCAGCC
*DKK2*	ENSCAFG00000011087	AGAGATCGAAACCATGGCCA	CCTTCTTGCGCTGTTTGGTA
*GALNT15*	ENSCAFG00000005887	TGGGGCACATCTACCGAAAT	CAGGGTAGATGTTGGCCAGA
*KANK3*	ENSCAFG00000029570	ATGGCCAAGTTTGCCCTGAAT	CAGCTCCTCCACGTACTTGA
*CCBE1*	ENSCAFG00000000093	CTGCCTGGATATCGACGAGT	TCCACCGCATTCTCAGACTT
*LYVE1*	ENSCAFG00000007580	AGTGCTTGCACTCCTCTTCT	TGGACTCTTGGGCTCTTCTG
*TMEM132C*	ENSCAFG00000006849	ACATCCTTGGAGCAGAGACC	TCCAGTTGAGGGAGAGCTTG
*BTBD11*	ENSCAFG00000001793	CCCCAAGCTCACAGAGATCA	AGTGCTTTGAACCTGGGAGA
*SLC37A2*	ENSCAFG00000010949	TTTTCACCTCGCTCTTTGGC	TACGGATGTGTGGGAGTTCC
*OLR1*	ENSCAFG00000013495	TGCTGTGACACTAGGGATCC	TCAAGCTTCTGGGTAAGGGT
*GDF6*	ENSCAFG00000009445	CCAACACCATCACCAGCTTC	CTGGGGATAATTCGAGGCCA
*B4GALNT3*	ENSCAFG00000015788	ATGACTATACCCGCCTGAGC	GGCCCTTGTTTTCCTTCTGG
*LRRN2*	ENSCAFG00000009675	AGCTCAACTACCTGGCCAAT	AGAGCTGGTTGTGGTTGAGA
*SFRP2*	ENSCAFG00000008353	AAGTTCCTCTGCTCGCTCTT	GGCTTCGCATACCTTTGGAG
*AK5*	ENSCAFG00000020380	GTCAAGGAATTAGGCGGCTG	TGGAGGAAGCCGATCATACC
*CXCL14*	ENSCAFG00000001084	CTACAGCGACGTGAAGAAGC	ACCACTTGATGAACCGCTTG
*PRLR*	ENSCAFG00000018782	TGGGCAGCAGACTCAGTTTA	ATGACAGCAGAAAGAACGGC
*MMP27*	ENSCAFG00000015066	TTCCCAAACCCATCCGTACA	CTGGAAAGCAGCATCGACTC
*PLXDC1*	ENSCAFG00000016530	ATGGCCAATTTCAACCCTGG	GGCGTCTGATAGTCCTGCTT
*RASGRF2*	ENSCAFG00000008744	TCCAAGAAGCCTCCCATCAG	CTTGGAGGCTGTACTGGTGA
*GLP1R*	ENSCAFG00000001547	GTGTTCCCCTGCTGTTTGTT	GACACCACGATGCAGATGAC
*KIAA1755*	ENSCAFG00000008896	ACCCAGGACTCATCAAGGTG	AGGGGTGGCATTCTTTGAGA
*IGSF10*	ENSCAFG00000008537	ACGCACTGATTACTGTCCCA	TGGCCTCTCCCATGTGATTT
*COL6A6*	ENSCAFG00000006035	CTTCCGGGAGAGATGGGATC	TTCATGCGCTCGAATTCCTG
*HOXB9*	ENSCAFG00000016867	AAGTTTCCTTCCGGCCAGTA	GCAGGTAGGGGTGGTAGAC
*SLC4A10*	ENSCAFG00000010242	ATTGATGGTGGCTGTCATGC	AGTAACCCTTTGCTCCCGAA
*ALPK2*	ENSCAFG00000000107	GGCGAGTGGACATTTAGCTG	GAGGTCAGGGTACATCTCGG
*CASQ1*	ENSCAFG00000012470	GAGTTCTCTGCTGACACCCT	CCTCGAAGGCCTTGTAATGC
*SERPINE1*	ENSCAFG00000013909	CCTCCCCATTCTTCGGTCTT	TGCCCGAGTTTGGTAGGAAT
*ZBED2*	ENSCAFG00000030690	GAGGAGCTGGAGAAGACTGG	CTCCTGAGCACCTCCTTCTC
*CNN1*	ENSCAFG00000017326	AGTATGACCACCAGCGAGAG	TCGTTGACCTTCTTCACGGA
*ACTB*	ENSCAFG00000016020	TCGAGACTTTCAACACCCCA	CATGAGGTAGTCGGTCAGGT
*HPRT1*	ENSCAFG00000018870	CCCAGCGTCGTGATTAGTGA	AGAGGGCTACGATGTGATGG

## Data Availability

All of the data discussed in this work, if not already included in the manuscript, are available from the corresponding author on reasonable request.
